# Validating a framework for Pinnacle plan conversion and archival

**DOI:** 10.1002/acm2.70222

**Published:** 2025-08-21

**Authors:** Samuel D. Rusu, Caiden Atienza, Blake R. Smith, Joel J. St‐Aubin, Daniel E. Hyer

**Affiliations:** ^1^ Department of Radiation Oncology University of Iowa Iowa City Iowa USA

**Keywords:** gateway, pinnacle, ProKnow, TPS archive, TPS file conversion

## Abstract

**Purpose:**

The Pinnacle treatment planning system support ends after December 31, 2026. As access to patient treatment records is necessary for continued care, a validated method to access and use this historic treatment planning data is needed.

**Methods:**

Sun Nuclear Gateway was used to convert over 20,000 patients stored in the TAR format for 8 versions of Pinnacle and archive this data into DICOM formatted images, structures, and dose re‐sampled to 2 mm. Using this solution, a total of 800 patients were automatically converted and pushed to ProKnow, a commercial cloud‐based RT‐PACS. An initial quality check was performed to verify that the data was converted and associated correctly. Twenty‐five random patients were then validated for each version of Pinnacle by reviewing plan name, the MU per beam, structures, max dose point, the number of beams, and the number of fractions. For each Pinnacle version, a 1%/1 mm 3D gamma analysis between the Pinnacle exported and the Gateway converted RT dose for a random plan was performed. The amount of space and time required to archive Pinnacle plans to DICOM format was recorded.

**Results:**

The average patient Pinnacle TAR size was 687.38 ± 599.62 MB and required 120 ± 49s to archive with an average conversion size of 155 ± 26 MB. A 2% initial archival error was observed that was remedied by repushing the affected patients to ProKnow. A conversion error rate of 0.5% was observed for the described validation process for the 200 patients plans. The average 1%/1 mm 3D gamma analysis pass rate was 99.96%.

**Conclusions:**

Gateway has been validated as a conversion solution of TAR data to DICOM for Pinnacle versions 7.4f, 8.0d, 8.0m, 9.0, 9.2, 9.8, 9.10, and 16.2.

## 1 INTRODUCTION

Pinnacle^3^ TPS (Philips Radiation Oncology Systems, Fitchburg, WI, USA) historically was one of the most used treatment planning systems worldwide, with over 34% of the market share in the US and Canada.^1^ However, in recent years, clinical use has declined, which has led to Philips selling its patent portfolio to Elekta (Elekta AB, Stockholm, Sweden) and concluding Pinnacle support by December 31, 2026.^2^, ^3^ As a result, a seamless conversion and archival of Pinnacle data for existing Pinnacle users is needed. Our institution has utilized Pinnacle to treat over 20,000 patients across a 20‐year span using the following versions 7.4f, 8.0d, 8.0m, 9.0, 9.2, 9.8, 9.10, and 16.2. This data exists as TAR files organized in monthly folders on an IPSILON server. To automate this conversion, the Sun Nuclear Gateway (Sun Nuclear Corp, Melbourne, FL, USA) product was selected. Sun Nuclear Gateway (Gateway) can automatically convert native or TAR formats of Pinnacle files (versions–4.x to 18.x) to DICOM formats.^4^ The CT data, structures, and dose are converted to DICOM files, which are resampled at a 2 mm slice thickness from 2–4 mm in Pinnacle. The amount of space, time, and architecture needed to archive old patient plans in DICOM format is of interest to other clinics that will need to archive patients from the Pinnacle TPS for retreatment and legal requirements. Once this data is converted, it can be used as needed in Gateway or stored using an alternative archival system.

Our institution chose to archive the converted Pinnacle data on the ProKnow DS (Elekta AB, Stockholm, Sweden) system. ProKnow DS is a cloud‐based RT‐PACS (Radiation Therapy Picture/Patient Archiving and Communication System) designed to be used as an accessory system for patient data archiving, information management, and analytics. This platform is convenient to use for quick archival retrieval and potential data mining.

## 2 METHODS

**FIGURE 1:**
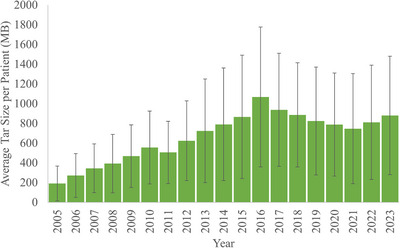
The average TAR size per patient over time (MB).

The scope of this conversion and archiving project is illustrated in Figure 1 by showing the storage space occupied by TAR files for each year that Pinnacle was used clinically at our institution, with their standard deviation. The average size of a patient TAR file was 687.38 ± 599.62 MB and required over 25 TB of hard drive space allocated for long‐term storage. File sizes varied by treatment based on the plurality of images registered to the planning CT, including 4DCT data or other imaging modalities.

**FIGURE 2:**
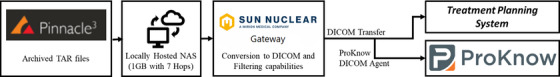
The architecture used for the conversion, validation, and archival of Pinnacle data to ProKnow.

A vendor‐neutral DICOM format architecture design was developed to convert Pinnacle TAR‐formatted files, which is shown in Figure 2, and includes a locally hosted workstation that was designated for conversion and intermediate storage of data. The Gateway application was installed on this workstation, and a 48 TB network‐attached storage (NAS) was integrated. The necessary size of this network storage can be inferred from the average patient file size, as previously mentioned. The NAS was used to store Pinnacle TAR files as well as converted patient data locally to the workstation prior to export to ProKnow. The secure copy protocol via the command line was used to transfer the Pinnacle data from the archived location on the IPSILON server to the NAS using a 1 GB per second (Gbps) network with seven hops between the two systems. During this archival, the time and storage required to transfer all patients were recorded. Once all the patient data was transferred to the local NAS, the patient data was indexed using the Gateway.

The indexing process was timed using a subset of 600 patients, where Gateway was used to scan the specified folder with TAR files, read the institution, patient ID, name, number of plans, Pinnacle version, size, and write to a file within the Gateway application. Gateway allows for searching, grouping, and filtering of these files to allow the user to find the data they require without having to convert all patients. Following successful indexing, patient data could be converted to a DICOM format.

To validate the DICOM conversion using the Gateway, one hundred patients were randomly selected from each Pinnacle version (800 in total), converted to DICOM format, and then pushed to ProKnow. The conversion time and resulting DICOM file size were recorded for all 800 patients. A ProKnow DICOM agent listener was configured and used to accept DICOM data and import the data into ProKnow. The DICOM push from Gateway enabled the use of custom filtering in which only the composite plan and dose were sent; RT plans with no dose, structure sets, or images that did not have an associated RT plan and RT plans whose names consisted of “phantom”, “not used”, “copy”, “DNU”, or “QA” were filtered and not sent. The time for this DICOM transfer was recorded for each Pinnacle version.

An initial quality assurance check was conducted on the transferred data to ProKnow to ensure the data transfer was complete. The patients were sorted by the number of DICOM objects, and any patient that did not have at least four DICOM objects were manually flagged as not being properly transferred and were repushed. Each version of Pinnacle was individually pushed to ProKnow, and a CSV file was generated of all the patients in that workspace. This was compared to the patients that were pushed from Gateway, and if there were any duplicate patients (same patient but different plans), the patients were opened, and the data transfer was verified for all the patients that were pushed per version. This manual check verified that for all the patients that were pushed, the filter worked as intended and that the complete data was transferred to ProKnow. It also confirmed that the RT plan, dose, structure, and images were correctly associated.

Twenty‐five patients from each version of Pinnacle were randomly selected and validated against MOSAIQ and Pinnacle, when possible, to ensure accuracy and consistency between the conversions. Referencing the patient's MRN, each patient file was loaded in both MOSAIQ and ProKnow where the information recorded in the TPS notes (plan, trial, and prescription name) in MOSAIQ was matched against the information in ProKnow. The number of beams, fractions, and the monitor units (MU) per beam were validated by cross‐referencing the information in the ProKnow plan tab with the treatment record in MOSAIQ. Additionally, the isodose, structure sets, and CT images were reviewed to check that they were correctly imported and aligned properly. The global max dose for the plan in ProKnow was compared to the prescription in MOSAIQ to verify the magnitude of the converted RT Dose was reasonable. Lastly, one random patient's plan for each Pinnacle version was also verified using a 1%/1 mm 3D gamma analysis between the RT Dose pushed from Pinnacle and the Gateway converted RT Dose to detect any systematic errors in this automated process.

## 3 RESULTS

**FIGURE 3:**
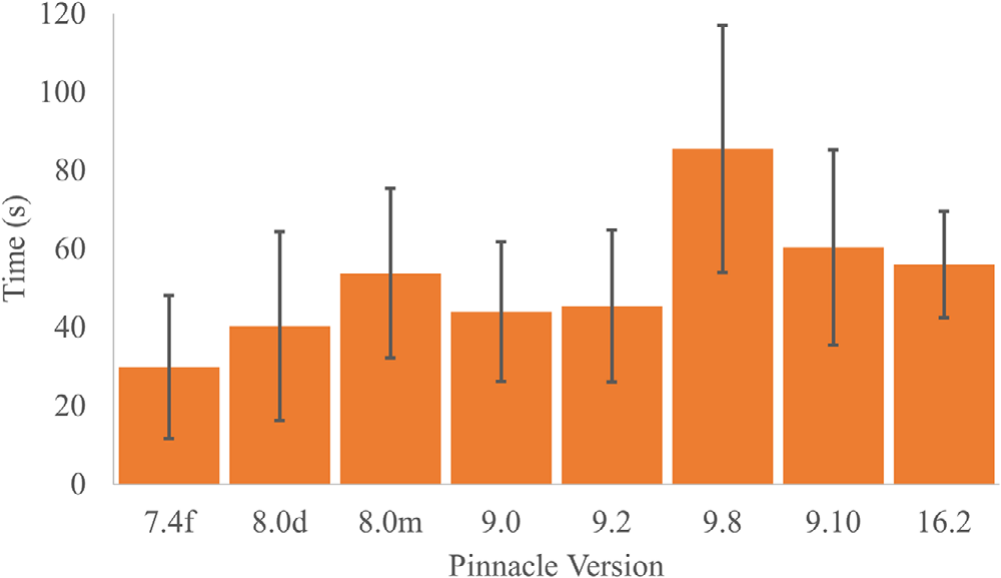
The average conversion time per patient categorized by Pinnacle version.

**FIGURE 4:**
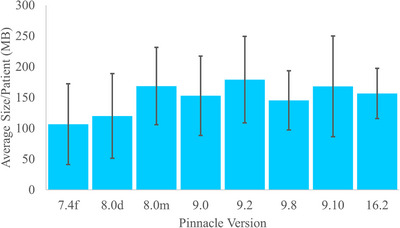
The average conversion size per patient categorized by Pinnacle version.

The transfer and conversion processes were evaluated using file size, transfer time, conversion time, and error rate. To transfer the TAR files from Pinnacle to an internal NAS over a 1 Gbps network with seven hops took an average time of 16 ± 1.0 s per patient. This corresponds to approximately a 2.58 ± 0.18 GB/min transfer rate. The indexing in Gateway for the patient's conversion took 6 ± 4s per patient. The conversion of the TAR files to DICOM took an average of 52 ± 21s per patient, with an average DICOM file size of 155±25.5 MB, which was much smaller than the original TAR file size of 687.38 ± 599.62 MB. The distribution of patient plan conversion times and file sizes are plotted in Figures 3 and 4, respectively, for each Pinnacle version, showing their average values and standard deviations.

**FIGURE 5:**
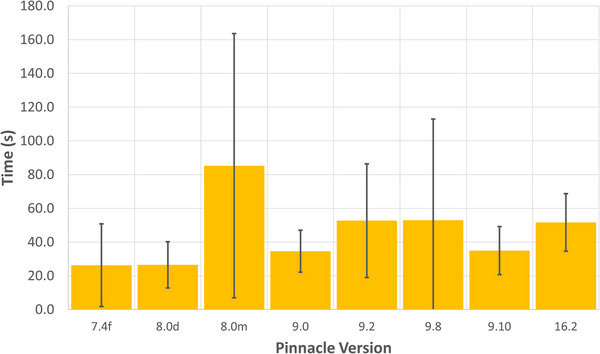
A representative of the DICOM average transfer time from Oncospace to some other DICOM software per patient categorized by Pinnacle version.

Transferring the converted DICOM files to ProKnow took an average of 46 ± 44s per patient. Although ProKnow was used for this work, the time presented in Figure 5 with its standard deviation is representative of the DICOM transfer time from the Gateway to a DICOM listener for each version of Pinnacle validated. A large standard deviation was observed in this data and was contributed to multiple factors, including network speed differences during the day, the number and type of imaging studies used for a particular treatment plan. The overall average total conversion and archival time from Pinnacle to ProKnow was found to be 120 ± 49s per patient. The post‐indexing time was found to be 98 ± 49s and gives an estimate of how long the DICOM conversion and push to the desired location will take if the Gateway is used as an on‐demand service. This information is summarized in Table 1.

**TABLE 1:** Summary of average time and size requirements per patient.

	Average Time (s)	Average Size (MB)
**Average Pinnacle TAR file size**		687.38 ± 599.62
**Transfer from Pinnacle to NAS**	16 ± 1	Estimated: 2.58 ± 0.18 GB/min
**Indexing time**	6 ± 4	
**Average conversion per patient**	52 ± 21	155 ± 26
**DICOM transfer time**	46 ± 44	
**TOTAL**	120 ± 49	155 ± 26
**Post indexing**	98 ± 49	155 ± 26

During our initial quality assurance, an archival error rate of approximately 2% was observed among the patients reviewed. These errors occurred during the transfer of data from the Gateway to ProKnow and were primarily due to incomplete data transfers or an incorrect association of plan data. Many of these errors were easily remedied by repushing the DICOM data of the plan. Other errors were mitigated by working with Gateway to override the Pinnacle MRN with the CT DICOM MRN for a subset of unique instances were the same patient had multiple MRNs. In only one case, a patient's data was not converted for version 8.0d of Pinnacle due to the Gateway's software not being able to apply the correct conversion transform to convert this plan. With the exception of this error, no errors were found in the subset of items that were checked as described in the methods.

Although no clinically significant differences were found when verifying the number of MU, some rounding differences were observed. The max difference in number of MU between the converted and planned MU was 1 MU for one beam per plan. The average 3D gamma pass rate for the analyzed dose distributions was 99.96%.

## 4 DISCUSSION

There are multiple solutions available for a Pinnacle TAR file conversion. These solutions range in complexity of use and automation possibility when attempting to batch covert many patients. Solutions based on the PyMedPhys project and personal in‐house code exist or can be generated, but a commercial solution with support was preferred.^6^ As a result, the Gateway solution was chosen and validated.

In the subset of 200 patients that were validated, no conversion errors were identified except for the one patient whose data was not converted. For older versions of Pinnacle, during the TAR file conversion, Gateway must apply a complex transform to align the CT and associated data. If this fails, an error message from the Gateway is produced. Due to batch conversion and transfer, this error was not realized until it was validated in ProKnow. As a result, log files were analyzed in the Gateway to prevent DICOM from pushing unsuccessfully converted plans for the rest of the population of patients. There was no recovery from this error, and the patient dose and structure set was not converted. This error may be addressed in newer versions of Gateway, but our experience indicates that older versions of Pinnacle may result in a 0.5% conversion error that cannot be addressed without using Pinnacle to unarchive and DICOM export the data. Another limitation to note is that Gateway can only convert archived computed doses in Pinnacle. If plans were archived without the computed dose, Pinnacle must be used to convert this data.

During the initial quality assurance process, approximately 2% of plans were found to have an archival error linked to incomplete or incorrectly associated image datasets in ProKnow. Further investigation revealed that this random error occurred during the transfer from Gateway to ProKnow and could be remedied by re‐DICOM pushing the plan. For all these instances in which this error occurred, re‐pushing addressed the problem, and complete data transfer was observed.

A unique systematic error was identified that involved the incorrect association of plan data and imaging data when DICOM pushing from the Gateway. This issue was caused by a mismatch between the DICOM medical record number (MRN) in the imaging data and the MRN in Pinnacle. This error was limited to older patient records created before the department transitioned to a paperless system. This error was remedied by working with Gateway to override the Pinnacle MRN with the CT DICOM MRN. One limitation of this solution is that the correct patient MRN used in MOSAIQ could have corresponded to either the CT DICOM or Pinnacle MRN. This issue was not observed for patients with complete digital RO records.

The converted DICOM storage requirements were significantly less than the original compressed TAR format, primarily due to the limited archival of image datasets. In the current version of Gateway, multiple imaging information can be converted, but the current version does not save the registration between the images. As a result, Gateway was used to convert only the planning CT, associated structures, treatment plan, and dose, while supplemental image information remains accessible from the local PACS. This data is sufficient for performing EQD2 calculations and for re‐treatment dose evaluation.

The initial data transfer to the NAS via secure copy protocol was done separately for each calendar year. The total personnel time for this process was about 2 hours. Once all the patient data was on the NAS, the indexing was automatic in the Gateway software and only required manually selecting the folder that the data was in. No additional personnel time was required during this step, with the exception of a few minutes to initialize this process. However, converting the data in the Gateway software requires manual selection of the patients to be converted. The process was nearly instantaneous for less than 100 selected, while groups of 1000 or more required between 5–10 min to start the conversion process, which is automatic and requires no additional personnel time once started. The DICOM push to the desired location is also automatic. Validation and error investigation were the most time‐intensive tasks, requiring over 60 h to resolve individual errors and manually validate the conversion accuracy among the different versions of Pinnacle for the subset of patients investigated.

Given the potential value of large radiotherapy datasets and the need for convenient accessibility, our institution converted and archived all patient plans that were stored in Pinnacle.^6^, ^7^ After the initial data transfer to the NAS and indexing, an alternative workflow could involve converting patient data only as needed. With this approach, users would need access to the workstation with the Gateway, but this process would eliminate the need to dedicate weeks or months to an archival process. The conversion and DICOM transfer time were found to be approximately 98 ± 49s per patient post secure copy protocol and indexing. ProKnow DS was chosen as the archival location for the patient data since it provides a fast interface and tools to make use of big data. It also allows for automatic renaming rules for structures, the extraction of metrics of interest, and acts as a data repository and analytics system, which others have used to improve the quality of radiotherapy nationally.^5^ With a population sample of more than twenty thousand patients over 20 years, this data could be valuable for future clinical applications.

## 5 CONCLUSION

Based on our architecture, patient data was converted, filtered, and stored in ProKnow, and the original full Pinnacle data is also backed up on the local NAS, indexed in Gateway. Gateway has been validated as a solution for the conversion of Pinnacle data to DICOM for versions 7.4f, 8.0d, 8.0 m, 9.0, 9.2, 9.8, 9.10, and 16.2. An average total time of 120 ± 49s for the archive process is required, with an average conversion size of 155 ± 26 MB per patient.

## AUTHOR CONTRIBUTION

All authors were part of the development of the manuscript, editing, drafting of the paper, and approved the final version to be published.

## CONFLICT OF INTEREST STATEMENT

Joel St‐Aubin reports honorarium and research funding from Elekta unrelated to this work. Daniel Hyer discloses a consulting relationship with Elekta and research funding from Elekta unrelated to this work. The remaining authors have no conflicts of interest to disclose.
